# THE INFLUENCE OF PERSONALITY ON MEMORY SELF-REPORT AMONG BLACK AND WHITE OLDER ADULTS

**DOI:** 10.1093/geroni/igz038.805

**Published:** 2019-11-08

**Authors:** Nikki Hill, Jacqueline Mogle, Sakshi Bhargava, Tyler Bell, Rachel Wion

**Affiliations:** 1 Penn State University, University Park, Pennsylvania, United States; 2 Penn State College of Nursing, University Park, Pennsylvania, United States

## Abstract

Personality traits, particularly neuroticism, have been associated with self-reported memory problems, but little is known regarding differences across racial groups. Community-dwelling older adults (n=425; M(SD) = 76.7(4.7) years; 62.6% female; 72.0% White) without cognitive impairment completed up to 11 annual comprehensive medical and neuropsychological examinations as part of the Einstein Aging Study. Multilevel modeling tested: 1) the association of neuroticism, conscientiousness, extraversion, openness, and agreeableness with three types of self-reported memory problems (frequency, one-year decline, and ten-year decline), and 2) whether these associations differed by race, specifically Black and White. Neuroticism predicted self-reported frequency of memory problems and perceived one-year decline when considered alone; however, this did not remain significant after including all personality traits. Conscientiousness influenced perceived ten-year memory decline in Black older adults but not White. Our findings suggest that the influence of personality on self-reported memory problems may not be consistent across racial groups.

